# A rigorous method for multigenic families' functional annotation: the peptidyl arginine deiminase (PADs) proteins family example

**DOI:** 10.1186/1471-2164-6-153

**Published:** 2005-11-04

**Authors:** N Balandraud, P Gouret, EGJ Danchin, M Blanc, D Zinn, J Roudier, P Pontarotti

**Affiliations:** 1 EA 3781, Evolution Biologique, Université de Provence, 3 pl. V. Hugo, 13331 Marseille Cedex 03, France; 2 INSERM UMR 639, Laboratoire d'Immunogénétique de la polyarthirte rhumatoïde, faculté de médecine la Timone, 13005 Marseille, France; 3 AFMB-UMR 6098-CNRS (U1 – U2) Glycogenomics and Biomedical Structural Biology, Case 932, 163 Avenue de Luminy, 13288 Marseille cedex 09, France

## Abstract

**Background:**

large scale and reliable proteins' functional annotation is a major challenge in modern biology. Phylogenetic analyses have been shown to be important for such tasks. However, up to now, phylogenetic annotation did not take into account expression data (i.e. ESTs, Microarrays, SAGE, ...). Therefore, integrating such data, like ESTs in phylogenetic annotation could be a major advance in post genomic analyses. We developed an approach enabling the combination of expression data and phylogenetic analysis. To illustrate our method, we used an example protein family, the peptidyl arginine deiminases (PADs), probably implied in Rheumatoid Arthritis.

**Results:**

the analysis was performed as follows: we built a phylogeny of PAD proteins from the NCBI's NR protein database. We completed the phylogenetic reconstruction of PADs using an enlarged sequence database containing translations of ESTs contigs. We then extracted all corresponding expression data contained in EST database This analysis allowed us **1/**To extend the spectrum of homologs-containing species and to improve the reconstruction of genes' evolutionary history. **2/**To deduce an accurate gene expression pattern for each member of this protein family. **3/**To show a correlation between paralogous sequences' evolution rate and pattern of tissular expression.

**Conclusion:**

coupling phylogenetic reconstruction and expression data is a promising way of analysis that could be applied to all multigenic families to investigate the relationship between molecular and transcriptional evolution and to improve functional annotation.

## Background

The "in silico" functional annotation of proteins generated by large scale sequencing projects is an important challenge in biology. Here we propose a rigorous protocol for multigenic families' annotation.

### 1/Importance of phylogenetic reconstruction

Because important functions are conserved during evolution, the first step in analysis is to determine homologous sequences. More specifically, orthologs are more likely to share the same function while paralogs can undergo functional shifts. Developments and improvements in database similarity search programs such as BLAST [1] allowed the rapid identification of potential homologous sequences and therefore allowed functional prediction of several thousand of genes and proteins present in databases. However, the closest BLAST is often not the nearest neighbor [2]. Indeed, similarity-based approaches do not consider all the information from comparative and evolutionary biology. They do not differentiate between orthologs and paralogs among homologs. So, phylogenetic approaches, taking into account duplication and speciation events are necessary to robustly produce functional annotation of new, uncharacterized proteins [3-8].

### 2/Necessity to enlarge databases

For proteins' phylogenetic reconstruction, protein databases containing the proteomes of completely sequenced species along with individually submitted protein sequences are usually used (Ensembl protein db, NCBI Protein db etc...) [9,10]. Yet, the vast majority of species are not fully sequenced and most of their protein sequences are still unknown. However, a lot of transcriptional information is carried by growing gene expression databases, concerning normal or pathological tissues (Expressed Sequence Tags from NCBI, TIGR, GeneNote, Gepis etc...) [11-14]. These mRNAs could be used for (total or partial) reconstruction of unknown proteins in "not yet sequenced" species. In parallel, translation of EST contigs can be used to enlarge the spectrum of species containing homologs when one analyses a protein family.

### 3/Importance of expression patterns' determination for an accurate annotation

It should be noted that phylogenetic analysis can only give information at the biochemical function level. Furthermore, while orthologs can have very similar "molecular function", they can exhibit different "macroscopic functions", due to a transcriptional shift for example. To produce an accurate proteic functional annotation, one must have complete sequence information given by phylogenetic reconstruction, with expression patterns analysis. This is the second reason why using data from expression databases is interesting. Analysis of expression divergence between paralogs and orthologs have been recently published in Human and Mouse. It appears that gene expression profiles diverge between paralogs. Orthologs can diverge in their expression pattern too [15]. Moreover, orthologs that have undergone recent duplication have less strongly correlated expression profiles than those that have not [16]. Up to now, there is no study examining expression divergence between homologs that takes into account a broad spectrum of species and after a phylogenetic reconstruction.

### 4/Our approach

We present here a new way to functionally annotate proteins in silico, taking into account all these concepts. In a first step, we reconstructed the phylogeny of a protein family, using an enlarged database containing 1/full length proteins from NCBI NR protein database [10] and 2/translation of EST contigs from NCBI dbEST database [11]. We used a new software platform, FIGENIX [17], adapted to this kind of phylogenomic reconstruction. In a second step, we created an automated pipeline to couple these phylogenetic reconstructions with expression pattern data. We then compared the evolution rate of the different paralogous sequences with their patterns of expression.

We validated this approach with the peptidyl arginine deiminase multigenic family. These genes encode peptidyl arginine deiminase proteins (PADs) that are implied in Rheumatoid Arthritis (RA), a systemic autoimmune disease that primarily manifests as a chronic symmetric polyarthritis which gradually destroys articular cartilage and bone [18,19]. PADs transform Arginine residues into Citrulline in a calcium-dependant manner [20]. It was recently shown by a case-control linkage disequilibrium study that *PAD *type 4 is a susceptibility locus for rheumatoid arthritis in a Japanese population [21]. These data were not retrieved in a Caucasian population [22]. Up to now, no phylogenomic annotation coupled with expression data of this protein family has been proposed. We believe this analysis will shed new light on this incompletely characterized gene family.

## Results

### 1/Phylogenetic reconstruction (See material and methods for process)

**Figure 1:**
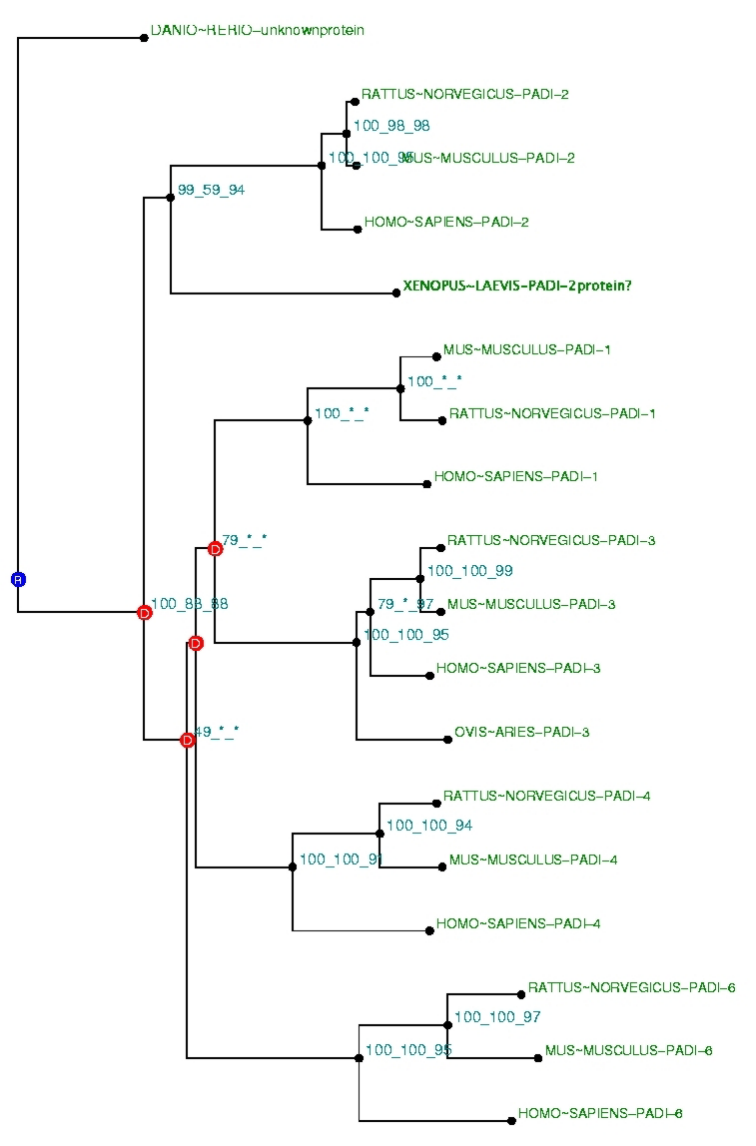
**Phylogenetic tree built with PAD and NR database, with Danio-rerio's PAD sequence in out-group**. This tree is the fusion on the NJ topology, of three phylogenetic trees built based on **N**eighbour Joining (NJ) [36], Maximum **P**arsimony (MP) [39], and Maximum **L**ikelihood (ML) [40] methods. The tree is labelled npl-A at the first root (for **N**j, m**P**, m**L**, with **A**ll topology congruence tests passed). For each node, bootstrap values are reported for each npl method. A "*" means that the bootstrap value was under 50%. PAD protein from Danio-Rerio is co-orthologous to all others PADs. In mammals five groups of orthologs are found, named PAD-1, PAD-2, PAD-3, PAD-4 and PAD-6. PAD-2 paralogy group is the best conserved as it evolved slower than the others.

First, we built a phylogenetic tree with full length PAD proteins (from NCBI NR protein database) (Figure 1). We found five paralogs of PAD proteins (types 1, 2, 3, 4, 6) encoded by five different genes as previously described [23]. We found PAD members in Birds (*Gallus gallus*), Amphibians (*Xenopus laevis*) and Teleosteans (*Danio rerio*). This tree supported by high bootstrap values suggested the following steps for evolution of *PAD *genes in craniates: *PAD*s' common ancestor was present as far back as in the last common ancestor of Teleostean and Mammals. *PAD-2 *first diverged by duplication from the common ancestor before the radiation of mammals.

**Figure 2:**
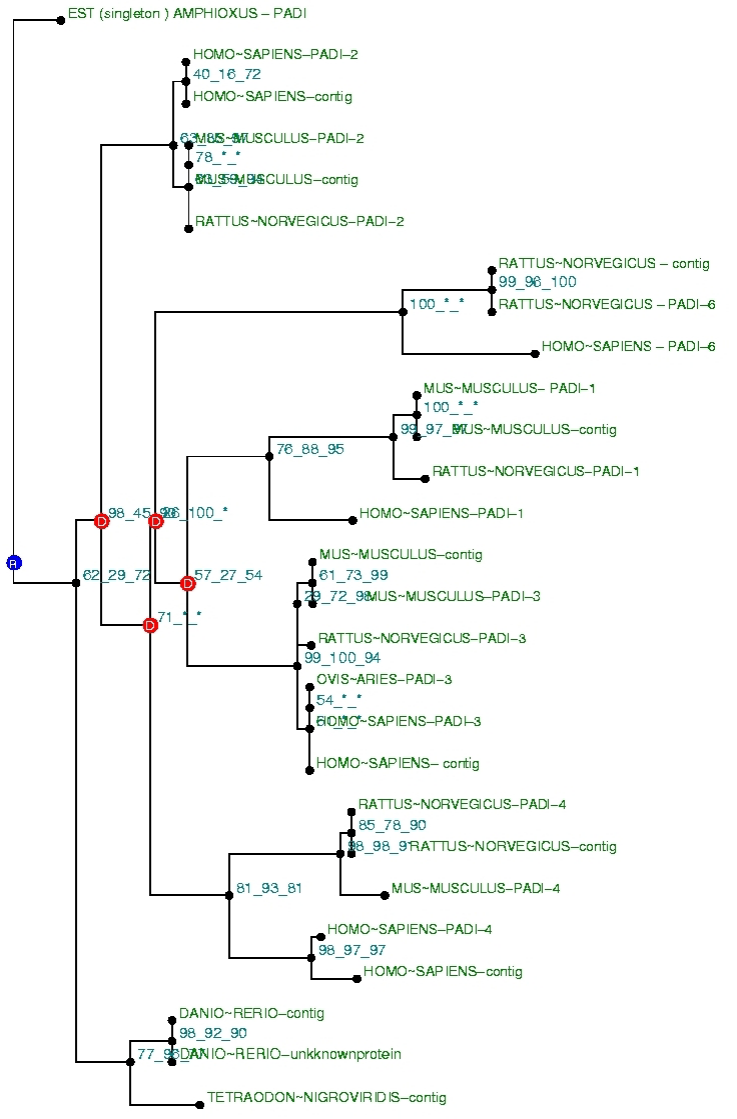
**Phylogenetic reconstruction with translated ESTs singleton**. One of the phylogenetic trees built with PAD and enlarged database (NR database and local translated EST contigs database). The tree was built with translated EST from PAD Amphioxus as query sequence, which was not included in contigs and in the five arbitrary domains. Building method is the same as in Figure 1.

We next built a phylogeny with the same protein dataset completed by additional proteins built from translations of EST contigs. First, BLAST searches on EST databases allowed us to identify other species in which PAD proteins are likely present. We found PADs in additional Teleosteans (*Fugu rubripes, Salmon, Ictularus punctatus, Onchorynchus-mykiss, Gasterosteus aculeatus*) and in Cephalochordates (*Branchiostoma Floridae*). Because ESTs are not full length sequences, they can not be used in this raw state to reconstruct a phylogenetic tree. To correct for this, we built larger sequences by merging ESTs relative to the same gene into contigs. EST contigs were translated and included in a local database and used for phylogenetic reconstruction along with protein from NCBI NR database. Because ESTs contigs often do not correspond to full length transcripts, we had to build several trees with alignment only on parts of the protein (five artificial domains). A tree built for each domain is available in additional file online [see additional file]. Some ESTs could not be integrated into a contig (these ESTs are called singletons) and were used in this state as sequence queries to reconstruct a phylogeny. An example of a tree built with a singleton (an Amphioxus sequence) is shown in Figure 2.

All the trees built with partial sequences were validated only if they were in agreement with the topology of the reference tree (Figure 1). This allowed us to classify all the contigs and singletons into paralogy or orthology groups. This analysis allowed us to propose a more detailed evolutionary story for the *PAD *genes: the common ancestor of the *PAD *gene was at least present in the common ancestor of the Euchordates (Cephalochordates and Craniates) (see tree of life in Additiona file online). It means that the *PAD *gene present in Amphioxus is co-orthologous to all PADs (as this is also the case for *PAD *genes in Teleostan). Analysis of trees' topologies allowed us to conclude that duplications of PADs certainly occurred after the speciation of craniata or more probably after the speciation of Sarcopterygian, but before the mammals' radiation. So, phylogenetic trees including translated EST contigs allowed us to enlarge the dataset of species and to classify these "new proteins" into a paralogy group.

When looking at the branches lengths, which are directly correlated with the sequence evolution rate, it appeared that some members of PAD genes evolved faster than the others. Branches lengths were longer within the PAD-6 paralogy group (Homo sapiens/Mus musculus/Rattus norvegicus PAD-6) whereas branches lengths were the shortest within the PAD-2 paralogy group (Homo sapiens/Mus musculus/Rattus norvegicus PAD-2). It simply means that the similarity between species is higher for PAD-2 than it is for PAD-6. This indicates that PAD-2s' group is the most highly conserved. Furthermore, the distance from the common ancestor is lower for PAD-2s' group than it is for the others, suggesting that PAD-2's group has been submitted to high negative selective pressure and has probably retained the ancestral biochemical function.

### 2/Coupling expression pattern with phylogenetic classification

**Figure 7:**
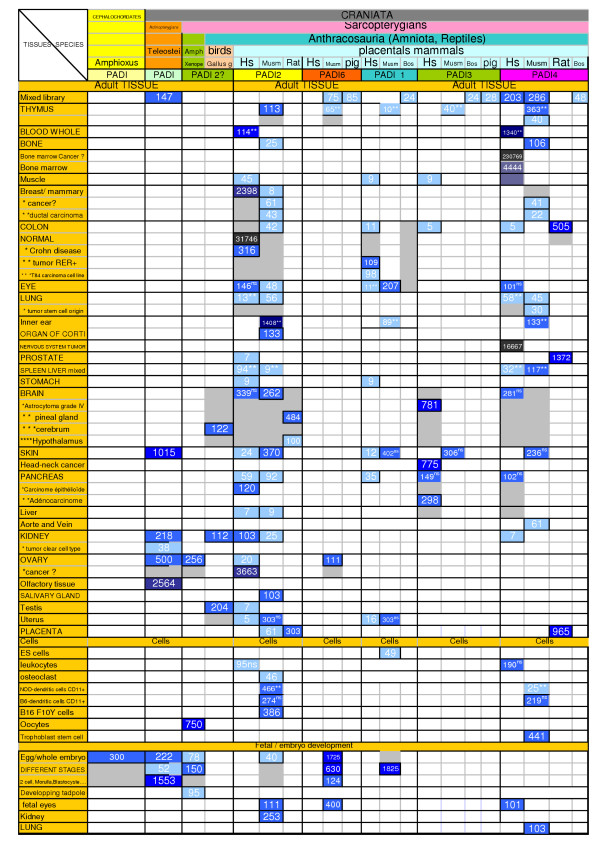
**PADs paralogs pattern of expression**. Proteins are classified as belonging to one of the four paralogy groups (*PAD *1, 2, 3, 4, 6) in columns. Lines correspond to tissue expression and cell categories. Numbers correspond to normalized number of hit (means of number of hit/number of clone × 1 000 000). Tissues are separated in adult tissues, cells and foetal tissues. In order to compare the expression levels from EST libraries between the different paralogous copies of *PAD *genes, we used the statistical test described by Audic and Claverie [25]. For each tissue type and cell category we compared the EST expression data (number of hits/number of clones in the considered library) for a given species between the different paralogous copies of *PAD *genes. NS: not significant. ** statistically significant difference.

The phylogenetic reconstruction allowed us to classify all the contigs or singletons in phylogenetic groups for the PADs phylogeny. We then developed software agents to extract transcriptional data (pattern of tissular expression) from the NCBI dbEST database [11]. We first normalized expression data (relative to the number of clones in each library), because expression data are not normalized in NCBI dbEST. We then built a table classifying ESTs according to their position in the tree (group of paralogs, orthologs or co-orthologs) and to the tissue in which they are expressed. Results are illustrated in a simplified Figure 7. Raw data are in Table 2 [see Additional file]. Given the current EST coverage, we focused on human and mouse. The bias could occur when, say, the coverage of the zebrafish is much lower than Human or Mouse. EST data are all represented in the table, but statistical analysis took into account only Human and Mouse data. For Homo sapiens and Mus musculus, data are congruent with expression patterns data available on UniGene [24].

Coupling phylogenetic classification with expression data permitted us to perform updated footprints of the transcriptional pattern for each paralogs group in this multigenic family. One group of paralogs, PAD-2, showed a broad tissular expression. As the expression pattern was different across paralogous *PAD *genes, we could only compare a few tissue types for differential levels of expression. The statistical test described by Audic and Claverie [25] was used. Significant differences (p > 95%) were shown in thymus, whole blood, lung, inner ear, mixed liver and spleen library and leucocytes (see Figure 7).

### 3/Coupling sequence evolution rate and expression pattern

**Figure 3:**
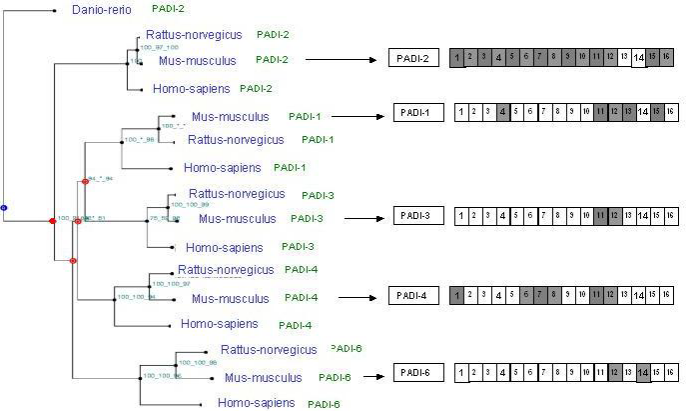
**Phylogenetic relationships and expression profiles in PADs family**. This is a fusion tree of three methods (Neighbor joining, maximum parsimony and maximum likelihood). Bootstrap values are calculated for these three methods. Branches length is correlated to sequence evolution rate. Branches lengths between PAD-6 paralogy group members are longer than between PAD-2 paralogy group members. Numbers in small squares correspond to different adult tissues: 1/bone, 2/brain, 3/colon, 4/eye, 5/kidney, 6/liver, 7/lung, 8/mammary gland, 9/pancreas, 10/placenta, 11/skin, 12/thymus, 13/uterus 14/ovary, 15/inner ear, 16/olfactory tissue. Grey squares indicate the presence of expression in the corresponding tissue in updated analysis (January 2005). White squares indicate no expression has been found in this tissue. Red circles indicate duplication.

**Figure 4:**
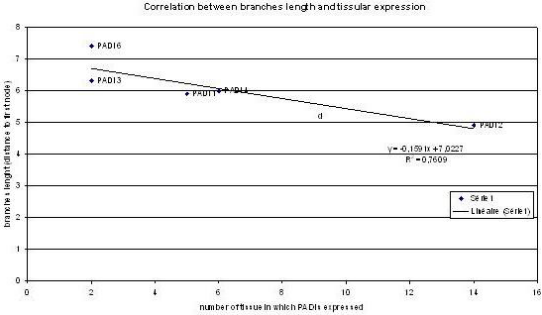
**Correlation between branches length and PAD tissular expression**: Branch lengths correlate with tissue expression as shown by the linear regression line. The coefficient of correlation R is 0.76.

When observing tissue distribution, we noticed that it was conversely correlated (Correlation coefficient R = 0.76) to sequence evolution rate (branches lengths) (see Figure 4). This was remarkable when we compared branches lengths and tissue distribution between PAD-2 (shortest branches, broadest tissue distribution) and PAD-6 (longest branches, more limited tissue distribution) (see Figure 3 and Figure 4).

## Discussion

### 1/Using phylogenomics and data from EST databases

Using EST data to complete data from protein databases is commonly performed. Yet, if one uses only the best BLAST hits approach to classify ESTs data, there is a large risk of misclassification into paralogy or orthology groups. Thus, expression data for a given paralogy group could be undermined by the wrong assignment of EST data into a group. In our example, we found additional expressed sequences from Teleostean and Cephalochordates in EST databases. When performing analysis with reciprocal best BLAST hit on NR protein database; EST from Amphioxus (cephalochordates) and Teleostean is classified as an ortholog of PAD-2 only, but not as a co-ortholog of all PADs as is shown by phylogenetic analysis. This illustrates why phylogenetic reconstruction is essential for accurate EST analysis and able to better avoid misclassification. Our approach enlarges the dataset of homologs-containing species and robustly and accurately classifies multigenic family members into paralogy and orthology group. Comparison of our results to those of Chavanas et al. [23], showed that our method allowed a more complete reconstruction of these genes' evolutionary histories, including a broader spectrum of species (such as cephalochordates). Our evolutionary scenario of duplications which occurred after the radiation of mammals is consistent with the co-localization of *PAD *genes in the same genomic region (1p35 in Human, chromosome 4E1 in Mus musculus and chromosome 5q36 in Rattus norvegicus), which suggests cis-duplications.

Notice that one third of reliably-inferred alternative mRNA isoforms are candidates for nonsense-mediated mRNA dacay (NMD), an mRNA surveillance system [26]. These transcripts, rather than being translated into proteins, are expected to be degraded and may be subject to regulated unproductive splicing and translation. In our study, we did not take into account this NMD-type alternative splicing, and took all information (assembling all exons) to reconstruct the most parsimonious story of a gene family. however, when looking at the overall data, we could suppose that some EST found in some tissus are not translated into protein but are regulatory elements.

Phylogenetic trees show that some PAD family members evolve faster than others, with the longest branches lengths seen in the PAD-6 paralogy group and the shortest branches lengths in the PAD-2 paralogy group. These data suggested that PAD-2s have probably kept the ancestral function and that other PADs may have run a neofunctionalization, still unknown. For example, the biochemical condition (Calcium concentration or pH) for enzyme activation could be different according to each PADs. Such an hypothesis has just been supported by Yamamoto's group who showed small different enzymatic properties between PAD-2 and PAD-4 [27].

Any kind of expression data could be used to enhance the accuracy of the analysis. The method used by Xun Gu et al. and developed in Genetics [28] and PNAS [29] is particularly interesting because they used microarray data in yeast to develop a statistical framework for studying the evolution of genes after duplications. On the basis of a Bayesian-based method, they reconstructed the evolutional trace of expression diversity after gene duplication. Their conclusions are in agreement with ours, showing that the expression of duplicate genes tends to evolve asymmetrically, one copy maintaining the ancestral expression profile while the others evolve rapidly.

2/When analyzing transcriptional data (Figure 7), we confirmed that transcriptional shift occurred between paralogs as previously shown [15,30]. PAD-2 shows a widespread expression whereas PAD-6 is expressed essentially during embryonic development. PAD-3, -4 and -1 have a restricted expression pattern (respectively thymus, eye, skin, ear and uterus for PAD-3, thymus, bone marrow, skin, breast, lung spleen, aorta and vein for PAD-4 and eye and skin for PAD-1). For orthologous genes, it is very difficult to compare different tissues in different species because 1/analysis is never done under the same experimental conditions and 2/one can never be sure that tissues evolved under the same physiological conditions in the different species.

3/Our analysis allowed us to correlate (R = 0.76) sequence evolution rate and tissular expression distribution of paralogs (figure 3 and 4). It is interesting to notice that shorter branches lengths are correlated with ubiquitous tissular expression. One can hypothesize that one copy (PAD-2) has probably kept the ancestral function, with ubiquitous expression. Other copies may have slightly different biochemical function, and underwent shifts in their expression pattern which is different and more limited. These data are consistent with studies showing that housekeeping genes evolve more slowly than tissue specific genes, and are under stronger specific constraints [31,32]. Our data suggest that the same phenomena could exist in multigenic families but, it must be confirmed in other examples.

## Conclusion

We believe our method of adding ESTs and expression data to phylogenetic analysis provides a new way to annotate multigenic families. More than classical phylogeny, it allows highlighting of transcriptional shifts between paralogs. It shows that functional shifts can occur in differential tissue expression rather than in biochemical function of the protein. It also shows a correlation between lower sequence evolution rate (branches length) and larger tissular expression distribution. For drug development, it points out the fact that when one analyses an orthologous protein in a species phylogenetically close to humans, one should keep in mind that tissular distribution of a protein can be different between species before extrapolation of the function to human.

This type of analysis is in its infancy and must be extended to other multigenic families and to all kinds of expression databases, including database where expression data are normalized such as in UniGene, SAGE and Micro arrays. In the future, the availability of more and more sequence information from different species will enable tracing a genome's evolutionary history, down to the expression data level, with more accuracy.

## Methods (cf. Figure 5)

**Figure 5:**
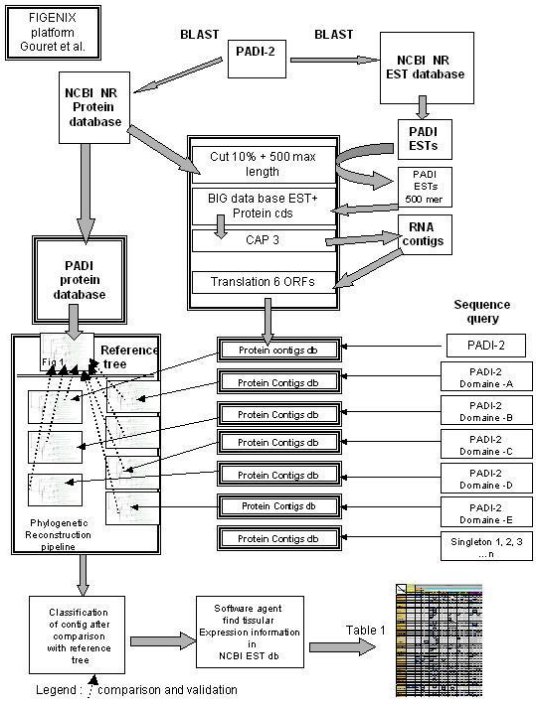
**Methods**. This picture illustrates the different steps of the software.

### 1-Phylogenetic reconstruction with full-length proteins, from NCBI NR database

PAD-2 was chosen as the query sequence for the first phylogenetic analysis. To build the phylogeny, the following steps, as described elsewhere [4] were used:

#### BLAST

Search BLAST hits on NR protein database [10] with e-value filter set to 1e^-4^.

#### Multiple alignment

Elimination of sequences disturbing alignments and doubles. Calculation of an alignment with CLUSTALW software [33]. Elimination of large gaps. Domains searching with HMMPFAM software [34]. Composition test for residues on Tree-Puzzle [35]. For each domain, elimination of non-monophyletic "repeats" in a tree built with NEIGHBOR JOINING algorithm on CLUSTALW software [36]. Elimination of sequences with divergent residue composition by using the amino-acid composition test from TREE-PUZZLE software [35].

Elimination of sites not under neutrality (fast evolving sites) by statistical methods for testing functional divergence after gene duplication [37].

Selection of congruent domains with HOMPART test from PAUP package [38], and building of a new alignment by merging preserved parts of domains' alignments.

#### Tree building

From this alignment, three phylogenetic trees were generated using the following methods: **N**eighbor Joining [36], Maximum **P**arsimony in Paup [39], Maximum **L**ikelihood in Puzzle [40]. By comparing topologies of these trees with PSCORE command (Templeton winning sites test [41,42]) from PAUP package and Kishino-Hasegawa test [43] from TREE-PUZZLE package, production of a fusion of these trees in a unique consensus tree according to the results of these tests.

Comparison of this fusion tree with the NCBI tree of life, , allowed deducing paralogous and orthologous proteins, using the algorithm described by Zmasek et al. [44]. Trees were rooted with the midpoint method. A fusion of the congruent three trees is noted **NPL-A **at the first root (for **N**j, m**P**, m**L**, with **A**ll topology congruence tests passed). Respective bootstrap values are noted in front of each node.

#### Analysis of the tree and rerooting

Our phylogenies are built with the midpoint method rooting. In order to avoid misclassification induced by fast evolving sites, sites which are not under neutrality are generally eliminated, using statistical methods for testing functional divergence after gene duplication [37]. In the phylogenetic "tree of reference", fast evolving sites could not be removed by Gu software because not enough sequences were present in the different paralogy groups. So, the PAD-6 paralogy group, which evolved faster than the others was automatically placed in out-group to equilibrate the tree. This was not necessarily reflective of the true evolutionary history of the PAD family. Indeed, when looking at the branches lengths between species belonging to the same paralogy group, particularly Mus musculus, Rattus norvegicus and Homo sapiens, we can deduce that duplication of the PAD ancestor occurred after the separation of Sarcopterygii and Actinopterygian. All of the species external to these groups in the tree of life were placed in out-group.

Indeed, if one placed PAD-6 in the out-group, the conclusion concerning PAD' evolutionary events would change. One would conclude that PADs duplicated before separation of Euchordates, but not after the emergence of Sarcopterigian. In this hypothesis, Cephalochordates and Teleosteans PADs reside in the PAD-2 paralogous group. This scheme is based on the hypothesis that PAD-6, -1, -3, -4 were all lost during evolution in Teleosteans and Cephalochordates. The tree built with PADI-6 in the out-group is farless parsimonious with regards to evolutionary events and is deducted from too many supposed gene losses. The same conclusions could be arrived at with other PAD groups placed in the out-group. So, a second tree, more parsimonious regarding evolutionary events was built with an out-group species rooting (Figure 1).

All this phylogenetic reconstructions were performed with the FIGENIX automated platform [17].

### 2-Building the tree including translations of ESTs contigs

In a second step, the tree was completed with sequences extracted from EST database.

#### BLAST

We first searched by BLAST request in NCBI dbEST database, sequences similar to PADs (result: 411 hits for e value 1e^-4^).

#### Creation of a local database

These short ESTs were put in a local database. This local database was then completed by mRNA (CDS) of completely sequenced proteins (NCBI protein db).

#### Contigs generation

Because ESTs are not full length mRNAs of known proteins, they cannot be included in a phylogenetic building in this raw state. We built a specific protein database using EST clustering. In order to reduce sequencing error, 10% of the EST sequence length was eliminated from each tip and only the 500 core central nucleotides were kept. Each species' EST group was analyzed with CAP3 (ESTs clustering software) [45] to produce RNA contigs (30 contigs constructed, 17 singletons). These RNA sequences were translated into proteins (6 open reading frames).

#### Phylogenetic reconstruction

Phylogenetic reconstruction was performed with the same method as described above. The phylogenetic analysis was at first built with the whole PAD-2 protein as the query sequence.

**Figure 6:**
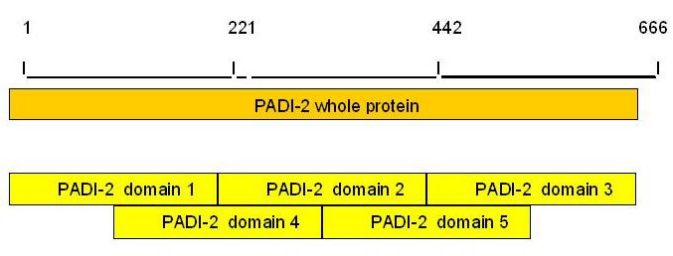
Five arbitrarily created domains of the protein PAD-2 (three equal sequences and two overlapping sequences). Each sequence was used as sequence query.

Some short sequences (not full length contigs) were eliminated during the phylogenetic process. For these sequences, phylogenetic trees were built using five arbitrary created domains of PAD-2 (three equal sequences and two overlapping sequences), used as the sequence query (Figure 6).

Using this method, most of the proteins translated from EST contigs could be analyzed and classified in a group of paralogs. However, 15 sequences were not overlapped by these arbitrary domains. These singletons were analyzed as sequence query in separated phylogenies. The trees were validated only if their topologies were similar to the topology of the reference tree (Figure 1). As an example, we provide a tree built with a protein from an Amphioxus PAD EST singleton (Figure 2). EST contigs incorporated in the trees are grouped with complete sequences (ex: contig: 14-1 in Mus musculus was close to complete PAD-3 protein of Mus musculus. It means that contig 14-1 contains ESTs from PAD-3 protein). Five trees built with this method are available on the supplementary material on line.

#### Transcriptional pattern analysis

Phylogenetic analysis allowed classification of each EST into a paralogy group. Information concerning tissue or organ provenance is given for each EST in the NCBI dbEST database [11]. We developed software "agents" in FIGENIX platform to automatically collect information corresponding to each EST. This expression information is: ESTs content of each contigs and tissue or organ expression corresponding to each EST. These data were pooled and classified in a summarizing table (Figure 7). For an accurate analysis of expression, we normalized expression data relative to the number of clones in each library (results are given in number of hit for 1 000 000 clones). Data were congruent with expression profile available for Homo sapiens and Mus musculus available in UniGene [24].

#### Differential expression significance and data consistency (See Figure 7 and Table 2 in supplementary material on line)

In order to compare the expression levels from EST libraries between the different paralogous copies of *PAD *genes, we used the statistical test described by Audic and Claverie [25]. For each tissue type and cell category, we compared the EST expression data (number of hits/number of clones in the considered library) for a given species between the different paralogous copies of *PAD *genes.

When a library was shared between two paralogous copies, we directly compared the two ratios. The threshold used for differential expression was set to p > 95% either for under or over expression.

When several different libraries were hit by a *PAD *gene for a given tissue or cell type, we first checked whether each library was consistent with all the other ones by applying the same statistical test [25]. When a given library was different from more than 50% of other libraries (using a threshold of p < 60% for over or under expression), we removed the significantly different library.

Only tissue and cells not associated with a pathological state were considered for comparison, as expression can be altered in cancers and other diseases. Similarly, genetically modified or selected mice variants were removed from the comparison as they may not reflect normal expression.

## Authors' contributions

Nathalie Balandraud initiated the study, collected the data and performed most steps of bioinformatic and statistical analysis presented in the paper. She developed the initial framework for the analysis of the EST genomic sequences. She produced all the phylogenies, all the Figures in the paper and Table 1, and wrote the manuscript. Philippe Gouret designed and supervised all the bioinformatic study with the creation of «Figenix» software. Etienne G.J. Danchin participated in creation of «Figenix» software, in the annotation of the gene set, participated in result interpretation, designed the statistical tests applied in the study and in writing later versions of the manuscript. Mathieu Blanc built the software agent to retrieve EST tissue expression data. Daniel Zinn co-participated in the creation of the software agent for ESTs. Jean Roudier participated in the inititation of the study and in writing latest version of the manuscript. Pierre Pontarotti supervised the study and co-wrote the manuscript.

## Supplementary Material

Additional File 1 Supplementary trees built with EST contigs. A simplified Tree of life. Table 2: normalized complete table Click here for file
