# Supplementary material for: A rigorous method for multigenic families' functional annotation: the peptidyl arginine deiminase (PADs) proteins family example
Source: BMC Genomics. 2005 Nov 4;6:153. doi: 10.1186/1471-2164-6-153 (PMC1310624; doi:10.1186/1471-2164-6-153)
Supplement: Additional File 1 — Supplementary trees built with EST contigs. A simplified Tree of life. Table 2: normalized complete table [file 1471-2164-6-153-S1.zip › suplemental data online/index.html]

PAGE Tree EST Contigs


**A rigorous
method for multigenic families**'
functional annotation:

the peptidyl
arginine deiminase (PADs) proteins family example

**N. Balandraud1\*, 
P. Gouret1\*,  E.G.J.  Danchin1,  M. Blanc1, 
D. Zinn1,  J. Roudier2,  P. Pontarotti1\*\***

  
  
  
  
  
    Supplementary
trees
built with EST contigs  
theses trees are built with an outgroup when it was necessary  
PADI-2 Domaine A-1-221  
PADI-2 Domaine B 222-442  
PADI-2 Domaine C -443-666  
PADI-2 Domaine D -110-331  
PADI-2 Domaine E -332-553
  
  
   Tree
of life

Table 2 : normalized complete table

Tree- A  
  
  
  
  
  
  
 
Tree- B  
  
  
  
  
  
  
  
  
  
Tree-C  
  
  
  
  
  
  
Tree D  
  
  
  
  
Tree E
