# Supplementary figures and images for: A rigorous method for multigenic families' functional annotation: the peptidyl arginine deiminase (PADs) proteins family example
Source: BMC Genomics. 2005 Nov 4;6:153. doi: 10.1186/1471-2164-6-153 (PMC1310624; doi:10.1186/1471-2164-6-153)

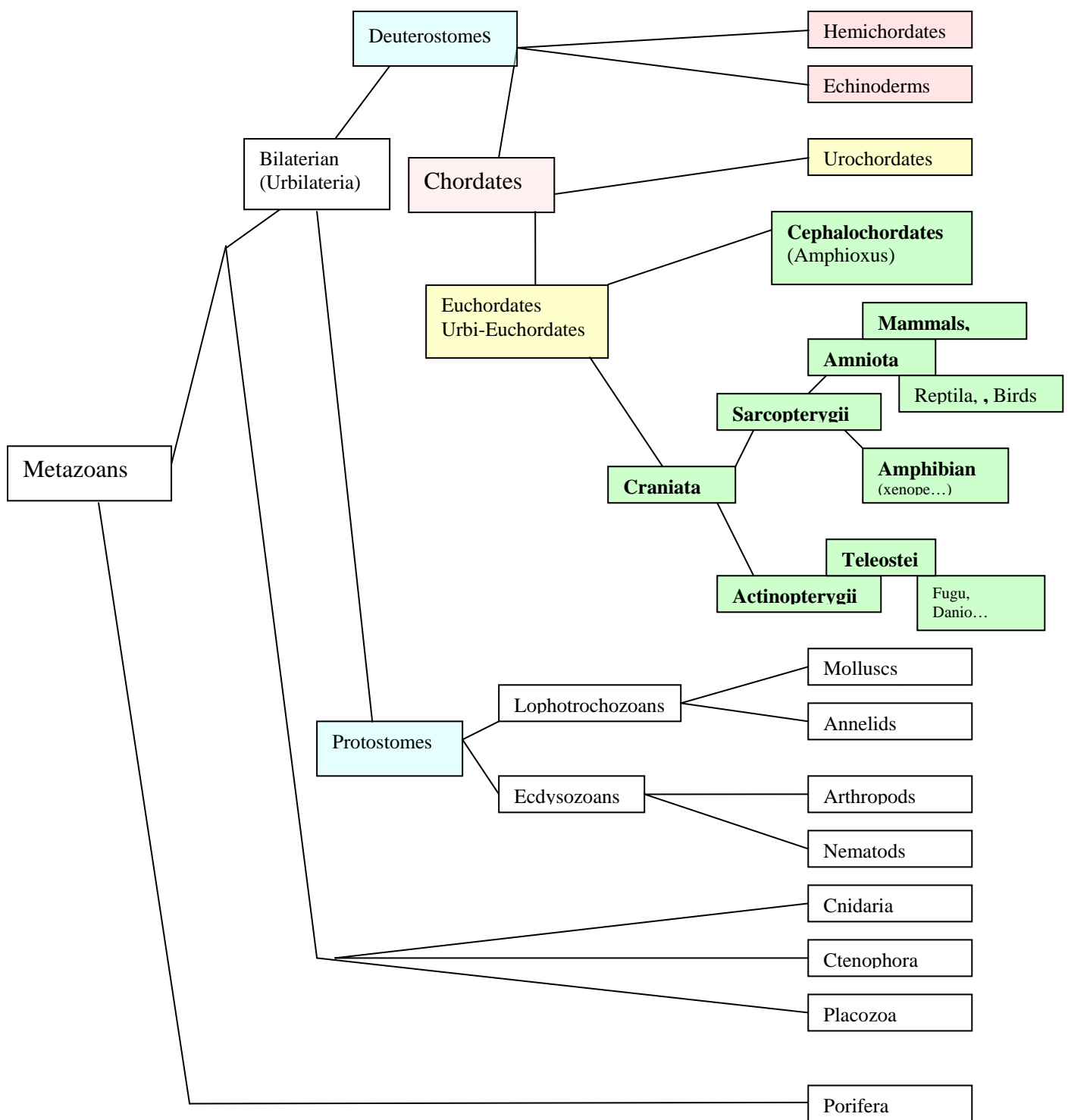

**Figure 1b : Very simplified tree of life. In green, species in which PADI protein were found**

Supplement: Additional File 1 — Supplementary trees built with EST contigs. A simplified Tree of life. Table 2: normalized complete table [file 1471-2164-6-153-S1.zip › suplemental data online/tree of life.pdf]

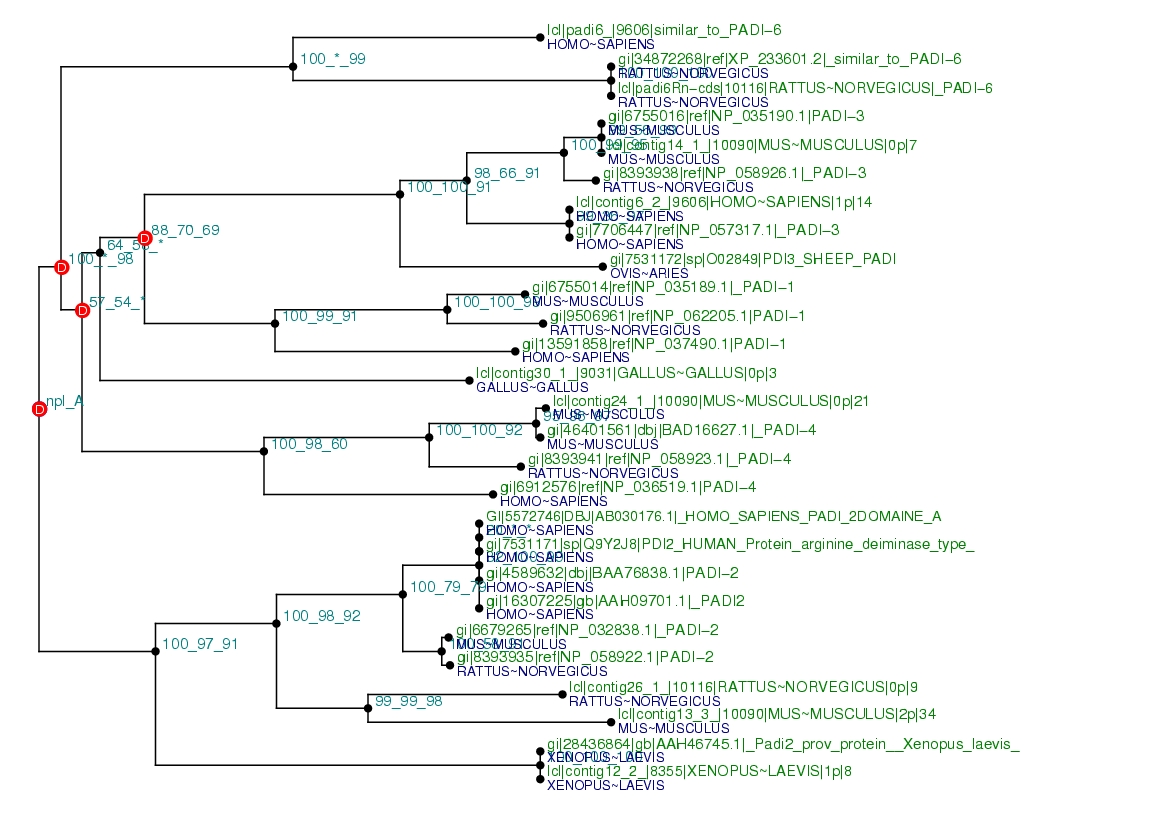

Supplement: Additional File 1 — Supplementary trees built with EST contigs. A simplified Tree of life. Table 2: normalized complete table [file 1471-2164-6-153-S1.zip › suplemental data online/Trees/Tree-A.jpg]

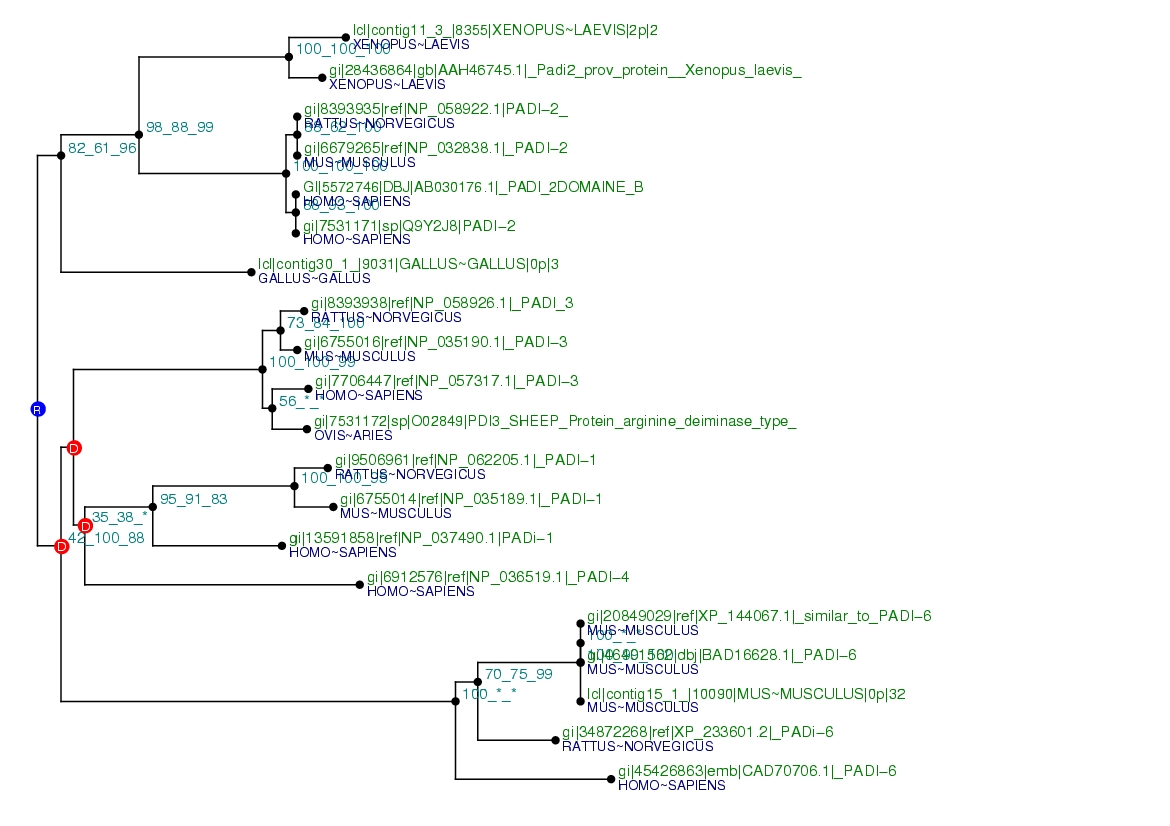

Supplement: Additional File 1 — Supplementary trees built with EST contigs. A simplified Tree of life. Table 2: normalized complete table [file 1471-2164-6-153-S1.zip › suplemental data online/Trees/tree-B.jpg]

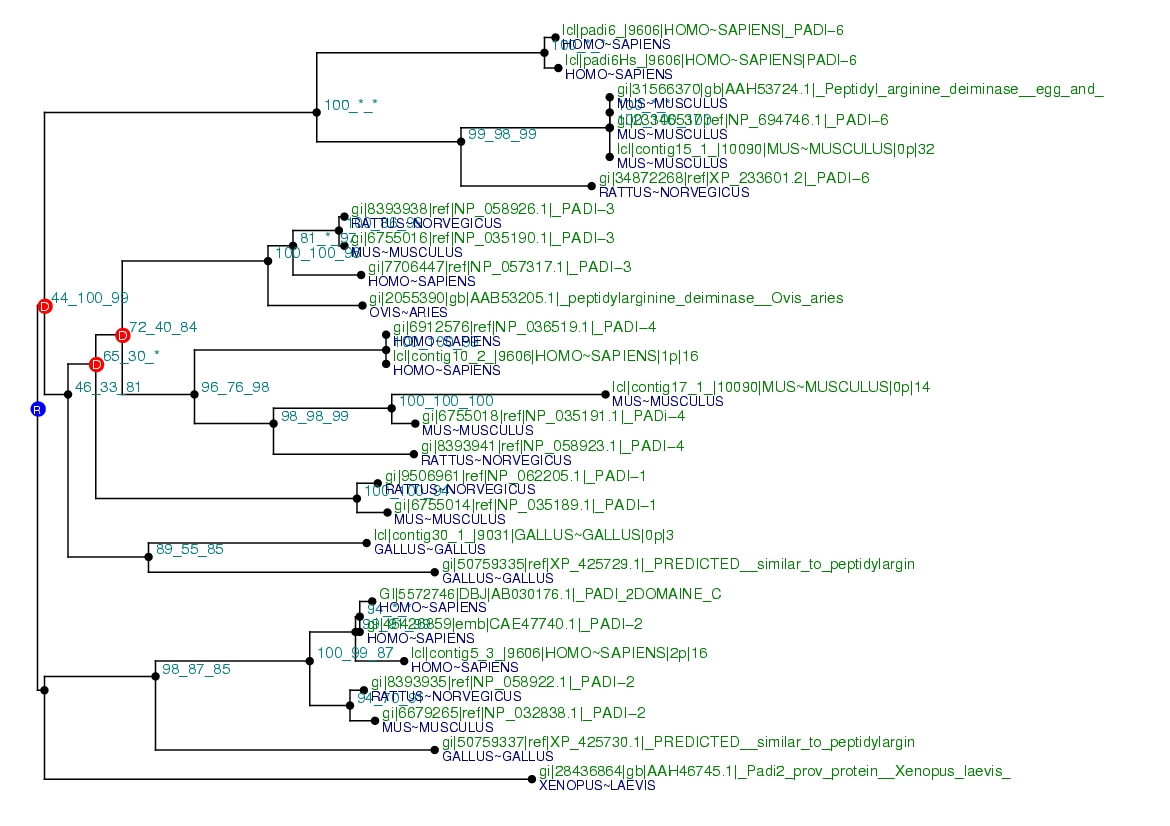

Supplement: Additional File 1 — Supplementary trees built with EST contigs. A simplified Tree of life. Table 2: normalized complete table [file 1471-2164-6-153-S1.zip › suplemental data online/Trees/Tree-C.jpg]

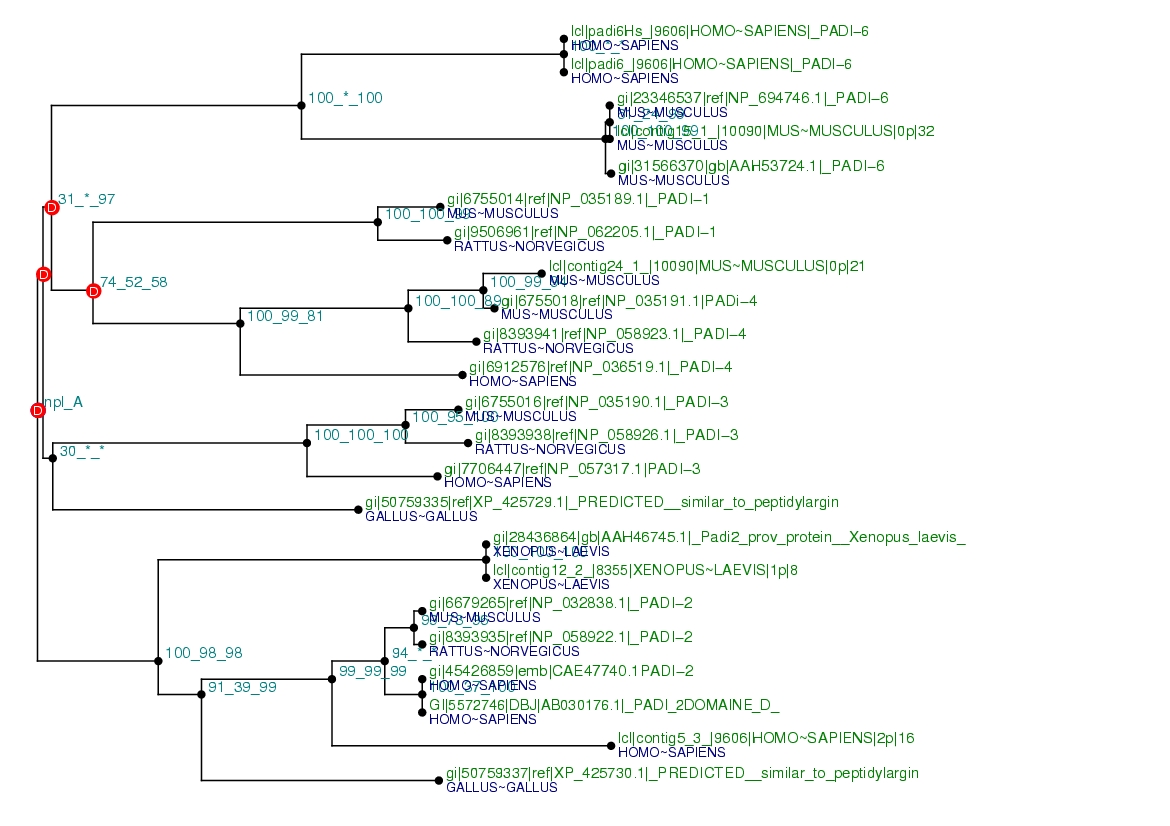

Supplement: Additional File 1 — Supplementary trees built with EST contigs. A simplified Tree of life. Table 2: normalized complete table [file 1471-2164-6-153-S1.zip › suplemental data online/Trees/Tree-D.jpg]

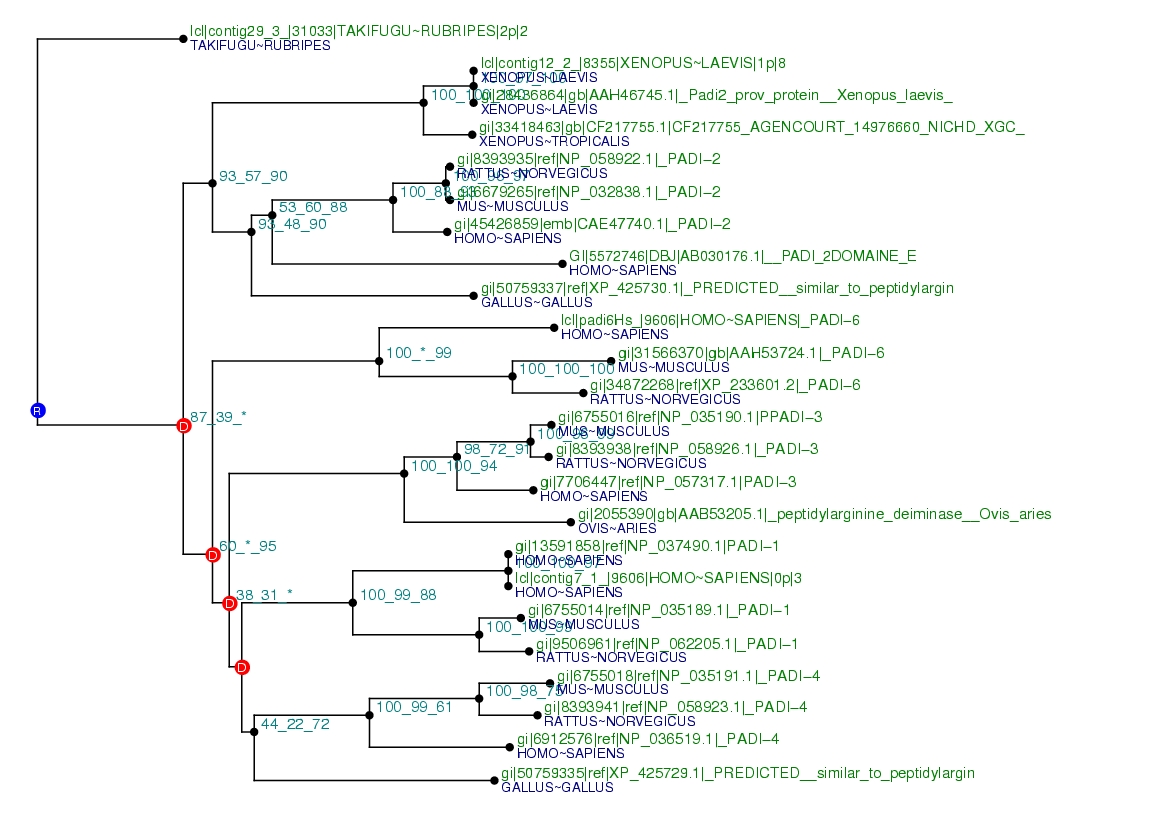

Supplement: Additional File 1 — Supplementary trees built with EST contigs. A simplified Tree of life. Table 2: normalized complete table [file 1471-2164-6-153-S1.zip › suplemental data online/Trees/Tree-E.jpg]
